# Transmembrane protease serine 5: a novel Schwann cell plasma marker for CMT1A

**DOI:** 10.1002/acn3.50965

**Published:** 2019-12-12

**Authors:** Hongge Wang, Matthew Davison, Kathryn Wang, Tai‐He Xia, Martin Kramer, Katherine Call, Jun Luo, Xingyao Wu, Riccardo Zuccarino, Chelsea Bacon, Yunhong Bai, John J. Moran, Laurie Gutmann, Shawna M. E. Feely, Tiffany Grider, Alexander M. Rossor, Mary M. Reilly, John Svaren, Michael E. Shy

**Affiliations:** ^1^ Translational Sciences Sanofi Research Sanofi Framingham Massachusetts; ^2^ Research Statistics Sanofi Research Sanofi Framingham Massachusetts; ^3^ Department of Neurology Carver College of Medicine University of Iowa Iowa City Iowa; ^4^ Waisman Center and Department of Comparative Biosciences University of Wisconsin Madison Wisconsin; ^5^ National Hospital for Neurology and Neurosurgery University College London London United Kingdom

## Abstract

**Objective:**

Development of biomarkers for Charcot‐Marie‐Tooth (CMT) disease is critical for implementing effective clinical trials. The most common form of CMT, type 1A, is caused by a genomic duplication surrounding the PMP22 gene. A recent report (Neurology 2018;90:e518–3524) showed elevation of neurofilament light (NfL) in plasma of CMT1A disease patients, which correlated with disease severity. However, no plasma/serum biomarker has been identified that is specific to Schwann cells, the most directly affected cells in CMT1A.

**Methods:**

We used the Olink immuno PCR platform to profile CMT1A patient (*n* = 47, 2 cohorts) and normal control plasma (*n* = 41, two cohorts) on five different Olink panels to screen 398 unique proteins.

**Results:**

The TMPRSS5 protein (Transmembrane protease serine 5) was elevated 2.07‐fold (*P* = <0.0001) in two independent cohorts of CMT1A samples relative to controls. TMPRSS5 is most highly expressed in Schwann cells of peripheral nerve. Consistent with early myelination deficits in CMT1A, TMPRSS5 was not significantly correlated with disease score (CMTES‐R, CMTNS‐R), nerve conduction velocities (Ulnar CMAP, Ulnar MNCV), or with age. TMPRSS5 was not significantly elevated in smaller sample sets from patients with CMT2A, CMT2E, CMT1B, or CMT1X. The Olink immuno PCR assays confirmed elevated levels of NfL (average 1.58‐fold, *P* < 0.0001), which correlated with CMT1A patient disease score.

**Interpretation:**

These data identify the first Schwann cell‐specific protein that is elevated in plasma of CMT1A patients, and may provide a disease marker and a potentially treatment‐responsive biomarker with good disease specificity for clinical trials.

## Introduction

The heritable peripheral neuropathies known as Charcot‐Marie‐Tooth disease (CMT) are the most common genetic neuromuscular diseases affecting 1:2500 individuals.^1^ The majority of CMT neuropathies are dominant and affect Schwann cell function and are classified as demyelinating (CMT1), although up to one‐third appear to be primary axonal disorders.^2^, ^3^ The most common form of CMT is CMT1A, which is caused by a duplication of the *PMP22* gene and subsequent overexpression of PMP22 mRNA and protein.^4^, ^5^ Animal models demonstrate that increased expression of PMP22 is sufficient to cause a demyelinating neuropathy, and that this can be reversed by reducing PMP22 expression.^6^, ^7^, ^8^ Accordingly, therapeutic approaches are currently being developed to decrease PMP22 expression.^7^, ^8^, ^9^ Investigating these upcoming therapies in patients, however, requires clinical trials based on clinical outcome measures and sensitive biomarkers.

Composite scoring systems have been developed based on CMT1A patients’ symptoms and neurological exam, termed the Rasch modified CMT Neuropathy Score (CMTNS‐R) and the Rasch modified CMTES (CMTES‐R).^10^ However, while the CMTNS has been used as a primary outcome in clinical trials,^11^, ^12^ annual change is small and at least several hundred patients would be needed in a double masked clinical trial to detect significant slowing of disease progression using either of these instruments as primary outcome measures.^13^ The progression of CMT1A results in muscle atrophy, and with neuroimaging, it has been demonstrated that the free intramuscular fat accumulation (IMFA) within calf muscles increases ~1–2% per year in patients with CMT1A.^14^, ^15^ The IMFA of muscle is specifically a measure of a myopathic or neurogenic disease process and is independent of a subject’s overall level of activity or fitness.^16^ These data suggest that a double masked trial in CMT1A could be performed with <100 patients/arm with MRI used as a primary outcome.

To complement the development of these outcome measures, there is a need to develop molecular markers of abnormalities in Schwann cells, degenerating axons or denervated muscle that could serve as biomarkers for severity and/or progression of CMT1A. A variety of muscle protein biomarkers have been identified in plasma or serum in muscular dystrophy studies,^17^, ^18^ and recent studies have indicated an elevation of neurofilament light in CMT1A patients (NfL or NEFL).^19^ To identify the potential molecular biomarkers in plasma for CMT1A, we obtained and analyzed samples from 47 subjects with CMT1A and 41 controls without known neurological disease, and analyzed them using five Olink Immuno PCR assay panels to identify significant differences in expression of targeted proteins. We also determined the correlation of the significantly regulated protein levels with disease score, nerve conductions, and age. These studies have revealed a novel blood protein biomarker that is preferentially expressed in Schwann cells.

## Methods

### Patient recruitment and consent

Institutional Review Board approval was obtained from University of Iowa and written informed assent/consent were provided by participants under a protocol approved by the ethics board of the NIH Rare Diseases Clinical Research Network (Protocol INC6601). Subjects with CMT1A were identified and evaluated in the Inherited Neuropathy Consortium (INC) clinic in the Department of Neurology at Iowa. Subjects were diagnosed with CMT1A on the basis of clinical evidence of sensory and/or motor peripheral neuropathy (including length‐dependent sensory loss, weakness and atrophy of the distal musculature, and decreased deep tendon reflexes), nerve conduction studies, and confirmatory genetic testing for the *PMP22* duplication in the subject or affected first‐degree relatives. Subjects and normal controls provided two 6‐ml EDTA‐containing tubes of blood during their visit for plasma extraction performed by standard techniques^20^ which was performed within 15 min of the blood draw and stored in aliquots stored at −80°C. All subjects were examined clinically by investigators who were certified by the INC for the proper administration of the CMTNSv2, a validated 9 item, 36 composite score based on patients’ symptoms (three items), examination findings (four items), and electrophysiology (two items).^21^ CMTESv2 scores were also calculated which included the seven items based on patients’ symptoms and examination findings in the CMTNSv2 but excludes the physiological results. Thus, the CMTES has a maximum score of 28 rather than 36 points.^21^ These scores were then subjected to Rasch modification to generate CMTNS‐R and CMTES‐R.^10^ Subjects with CMT1B, CMT1X, and CMT2A evaluated at the University of Iowa were also included and provided serum samples in an identical manner.

### Nerve conductions

Ulnar motor conduction velocities (MNCV) were performed by standard techniques^22^ with recording over the belly of the Abductor Digiti Minimi (ADM) with stimulation at the wrist and below the elbow. Maximum compound muscle action potential (CMAP) amplitudes were recorded using baseline to peak measurements in mV.

### Plasma assays

Aliquots from the plasma samples were screened by Olink immuno‐PCR profiling, by an in‐house developed Imperacer immuno‐PCR method for TMPRSS5, and by the Quanterix NfL methods. Olink (Uppsala, Sweden) profiling was performed using the manufacturer’s recommended protocols.^23^, ^24^ Initially, a subset of the samples was run on the Neurology Panel (92 proteins). Subsequently, the full set of samples was run on five Olink panels (neurology includes TMPRSS5 assay, neurology exploratory includes NfL assay, immuno oncology, immune response and inflammation panels [total 398 unique proteins]). Subsequent to the initial single Neurology Olink panel profiling, aliquots from the same samples were assayed by an in‐house developed Imperacer immuno‐PCR platform TMPRSS5 assay (Chimera Biotech; Dortmund, Germany) using the manufacturer’s recommended protocols and using R& D Systems anti‐human TMPRSS5 mouse monoclonal catalogue number MAB24951, and Goat polyclonal Catalogue number AF2495 antibodies. Quanterix NfL assays were performed according to the manufacturer’s protocols using the NfL‐Lite R kit on a single molecule array (Simoa TM) HD‐1 instrument (Quanterix, Lexington, MA).

### Statistical analysis methods

Data were analyzed using GraphPad Prism v7.03 (GraphPad, San Diego CA). Pearson correlation coefficients were calculated for each marker relative to neuropathy score (CMTES‐R, CMTES‐N, Ulnar CMAP, Ulnar MNCV). Correction for age effect was performed by partial correlation calculated using software package R v3.5.0 (Open source).

### AUC curve program

The TMPRSS5 and NfL ROC curves and AUC calculations were generated using R packages ROCR and pROC (R v 3.5.0 Open source).

### Cell culture experiments

S16^25^ rat Schwann cells were transfected with Sox10 siRNA (Ambion, 4390771) or control siRNA using Lipofectamine 3000 as described.^26^ RNA was isolated from three replicate cultures 48 h after transfection using Tri Reagent (Ambion) and relative levels RNA were determined quantitative RT‐PCR using the Comparative Ct method.^27^ Primers for *Tmprss5* and *Actb* were as follows: rTmprss5F: CAGTCTACTGTGCTGAGGAATG; rTmprss5 R: GAGACTTGCCCTGTATGTTAGG; rActb F: CACCCGCGAGTACAACCTTC; rActb R: CCC ATA CCC ACC ATC ACA CC.

## Results

To identify novel biomarkers for CMT1A, we analyzed plasma samples from those obtained from 47 subjects with CMT1A and 41 control subjects from two independent CMT1A cohorts and two independent control cohorts. The demographic details of each group are summarized in Table 1 (an additional four CMT1A cohort 2, and three control cohort 2 subject samples are included in the table which were included in a subsequent experiment looking at other CMT subtypes (Fig. 5). Control cohort 1 was taken from normal subjects without neuropathy, control cohort 2 was obtained from family members without neuropathy and not at risk for CMT1A or from volunteers without neuropathy from the University of Iowa. CMT1A cohorts 1 and 2 represent separate groups of patients with CMT1A. Demographic information concerning subjects with CMT1B (*n* = 17), CMT1X (*n* = 16), CMT2A (*n* = 4), and CMT2E (*n* = 9) is also provided. Age and gender distributions were similar in all patient and control groups. The mean Rasch modified Neuropathy and Exam scores (CMTNS‐R and CMTES‐R) were similar between the two CMT1A cohorts and also similar to what has been reported in the literature.^13^

**Table 1:** Demographic details, median TMPRSS5 and NfL plasma concentration, and Rasch modified CMTES and Rasch modified CMTNS of the CMT and healthy control cohorts.

Cohort group	N	Age, y (SEM)	Sex, F/M	TMPRSS5, NPX (IQR)	NfL, NPX (IQR)	CMTES‐R (SEM)	CMTNS‐R (SEM)	Ulnar CMAP (SEM)	Ulnar MNCV (SEM)
Control cohort 1	20	47.1 (2.8)	12/8	3.38 (2.96–3.62)	3.02 (2.57–3.14)				
Control cohort 2	24	49.4 (2.5)	14/10	3.42 (3.11–3.75)	2.96 (2.49–3.08)				
CMT1A cohort 1	20	47.4 (2.8)	13/7	4.48 (3.90–4.72)	3.63 (3.33–3.84)	14.20 (1.43)	18.80 (1.64)	4.36 (0.42)	21.45 (0.98)
CMT1A cohort 2	31	42.1 (2.8)	19/12	4.42 (4.09–4.74)	3.64 (2.95–3.97)	14.84 (1.16)	18.52 (1.59), *n* = 23	4.58 (0.47), *n* = 22	19.6 (1.09), *n* = 22
CMT1B	17	48.6 (4.9)	8/9	3.63 (SEM 0.10)					
CMT1X	16	47.0 (4.3)	6/10	3.63 (SEM 0.12)					
CMT2A	4	42.0 (4.8)	3/1	3.45 (SEM 0.26)					
CMT2E	9	46.9 (5.0)	5/4	3.44 (SEM 0.14)					

Abbreviations: CMT, Charcot‐Marie‐Tooth‐disease; CMT1, demyelinating CMT; CMT1A, 17p duplication; CMT1B, MPZ mutation; CMT1X, GJB1 mutation; CMT2, axonal CMT; CMT2A, MFN mutation; CMT2E, NfL mutation. Median numbers are given with SEM, standard error margin. TMPRSS5 and NfL median levels are given in NPX, normalized protein expression (Log2scale where a change of 1 is a twofold change) from Olink data; with IQR, interquartile range. CMTES‐R, Rasch modified CMTES Exam Score; CMTNS‐R, Rasch modified CMT Neuropathy Score; CMAP, Compound Muscle Action Potential; Ulnar MNCV, Ulnar Motor Nerve Conduction Velocity.

### Plasma proteins upregulated in Charcot‐Marie‐Tooth disease 1A (CMT1A)

**Figure 1:**
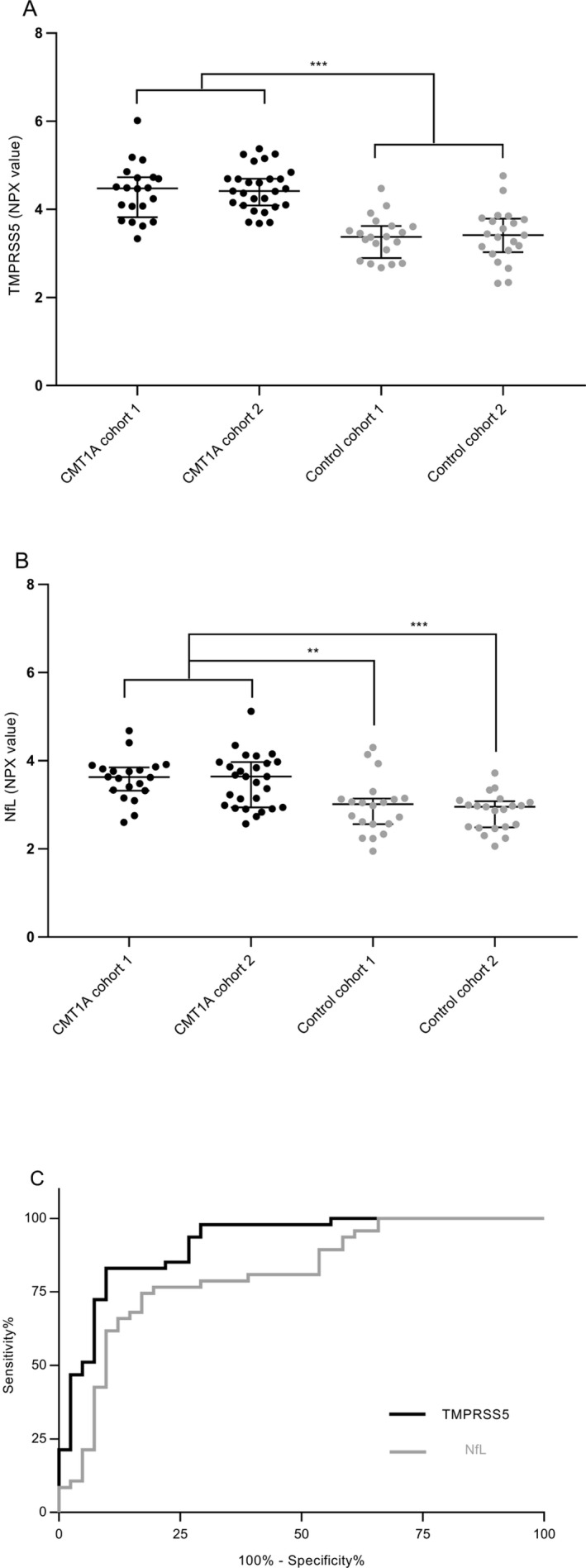
Elevation of TMPRSS5 in CMT1A plasma samples from multiple cohorts. Plasma samples obtained from CMT1A cohorts 1 and 2 and control cohorts 1 and 2, described in Table 1, were analyzed on Olink panels and TMPRSS5 (A) and NfL (B) levels determined, shown as NPX (a log2‐based parameter) with median and IQR. Comparisons by Anova four‐group comparison and Tukey’s multiple comparison test of the four groups. Then, both CMT1A cohorts, and both control cohorts were combined and compared by unpaired *t* test. CMT1A sample cohorts 1 & 2 with all control samples cohorts 1 & 2; of CMT1A cohort 1 with control cohort 1 or 2; and of CMT1A cohort 2 with control cohort 1 or 2, gave: TMPRSS5 fold change (FC): 2.07, 2.04, 1.99, 2.14, 2.08 and all *P* values < 0.001; NfL FC 1.58 *P* < 0.0001, 1.55 *P* 0.002, 1.70 *P* < 0.001, 1.49 *P* 0.003, 1.62 *P* < 0.001, respectively. No significant differences seen in comparison of CMT1A cohorts, or control cohorts with themselves: TMPRSS5 FC 0.95 *P* 0.975, 0.97 *P* 0.997; NfL FC 1.04 *P* 0.98, 1.09 *P* 0.877, respectively. Statistically significant elevation in comparisons of CMT1A cohorts to control cohorts is summarized as indicated ***P < 0.001, **P < 0.003–0.002. AUC plots are shown (C) for TMPRSS5 (area under ROC 0.9131, 95% confidence interval (CI) 0.8525–0.9736, *P* = <0.0001) and NfL (area under ROC 0.8085, CI 0.7157–0.9013, *P* = <0.0001).

An initial pilot screen of 15 CMT1A samples from cohort 1 against 10 control samples from control cohort 1 on the Olink Neurology panel had identified four candidate biomarkers that were elevated on average >twofold, *P* < 0.05 in CMT1A plasma, including TMPRSS5 (2.34‐fold *P* = 0.017), NMNAT1 (4.87‐fold *P* < 0.0001), LAT (2.03‐fold *P* = 0.0083), and MANF (2.75‐fold *P* = 0.0002) (Fig. S1). We then expanded the study by testing samples from both CMT1A cohorts 1 (*n* = 20) and 2 (*n* = 27), against control cohorts 1 (*n* = 20) and 2 (*n* = 21), on the five Olink panels (Neurology, Neurology Exploratory, Immuno‐Oncology, Immune Response, Inflammation [total 398 unique proteins]). In all, 339 of the proteins were detectable in >75% of samples tested, including 91 out of the 92 in the Neurology panel. We identified only TMPRSS5 to be consistently elevated in both cohorts of CMT1A samples compared to both control cohorts (threshold >twofold, *P* < 0.05) (Fig. 1A). TMPRSS5 was elevated on average by 2.07‐fold *P* < 0.0001 all CMT1A (*n* = 47) compared to all controls (*n* = 41), and also to comparable levels in the individual CMT1A cohorts compared to either control cohort. It should be noted that the Olink scale (NPX) is a log2‐based scale. TMPRSS5 is a serine protease that is also known as spinesin.^28^, ^29^

NfL was elevated on average 1.58‐fold *P* < 0.0001 for all CMT1A (*n* = 47) relative to all controls (*n* = 41), and to comparable levels in the individual CMT1A cohorts compared to either control cohort (Fig. 1B). This is comparable to the 1.76‐fold *P* = 0.0001 for the CMT1A subgroup in the previous report,^19^ measure using the Quanterix SIMOA assay. No other proteins on the five Olink panels tested exceeded this threshold of >1.5‐fold *P* < 0.05 for all the comparisons of individual CMT1A cohorts compared to either of the control cohorts.

The ability of TMPRSS5 levels to discriminate CMT1A patients from controls can be assessed using a receiver operator characteristic curve (Fig. 1C). The area under the curves was 0.913 for TMPRSS5 compared to a value of 0.81 for NfL (Olink data), demonstrating good specificity and sensitivity for discriminating TMPRSS5, and also NfL, from control levels.

To confirm the elevation of TMPRSS5 in CMT1A samples using an orthogonal assay, we developed an in‐house Imperacer immuno PCR assay using commercial TMPRSS5 antibodies and tested the same CMT1A cohort 1 and control cohort 1 samples shown in Figure S1. A similar upregulation of TMPRSS5 was observed, average 4.21‐fold *P* < 0.0029 (Fig. S2A), compared to the 2.34‐fold *P* 0.0017 observed by Olink analysis (Fig. S1). A very good correlation was observed between Olink and Imperacer TMPRSS5 levels *r* = 0.964 *P* < 0.00001 (Fig. S2B).

To confirm the accuracy of the Olink NfL assay, we determined the correlation between the Olink and Quanterix SIMOA assays in 10 CMT1A cohort 1 and 5 control cohort 1 samples. A very good correlation was observed *r* = 0.9735 *P* < 0.0001 (Fig. S2C).

### Correlation of TMPRSS5 and NfL with patient clinical data

**Figure 2:**
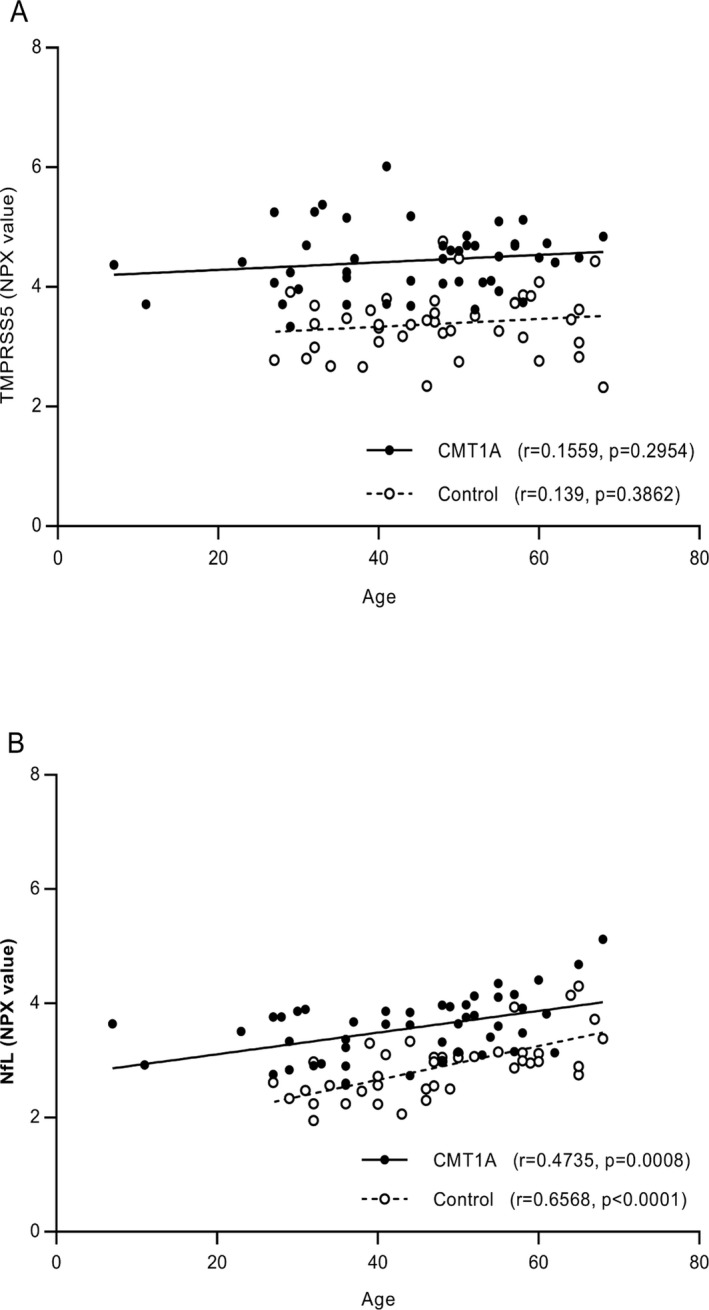
Correlation of TMPRSS and NfL with patient age. Correlations of TMPRSS5 (A) and NfL (B) levels with age (Pearson correlation coefficient) are shown for CMT1A and controls separately, with *r* and *P* values. The NPX value is a log2‐based parameter.

The correlation of TMPRSS5 and NfL with age was determined (Fig. 2). No significant correlation was found for TMRSS5 (Fig. 2A), whereas as expected,^19^ a positive correlation was observed for NfL for both controls and CMT1A samples (*r* = 0.6568 *P* = <0.0001 and *r* = 0.4735 *P* = 0.0008, respectively) (Fig. 2B). While the level was elevated in CMT1A compared to controls, the rate of NfL increases with age from the slope of the correlation plots in is similar for both (Fig. 2B). No significant sex effect was observed for the upregulation of TMPRSS5 or NfL in CMT1A samples (data not shown).

**Figure 3:**
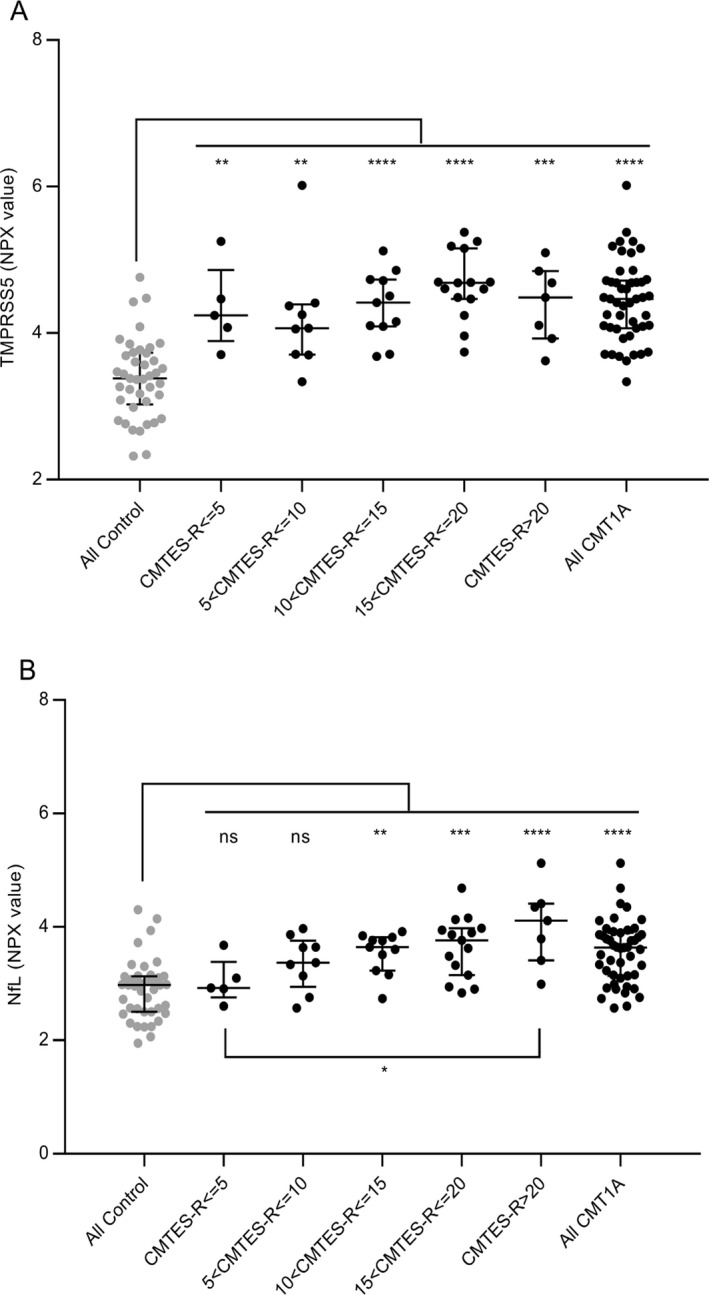
Elevation of CMT1A TMPRSS5 and NfL levels across binned Neuropathy Scores compared to controls. Elevation of TMPRSS5 (A) and NfL (B) with CMTES‐R neuropathy scores compared to controls, after binning CMT1A samples into five ranges from mild to severe (<5, 5 to <10, 10 to <15, 15 to <20, and >20) are shown (Tukey’s multiple comparison). Shown as NPX value a log2‐based parameter with median and IQR. For TMPRSS5 FC and *P* values across the bins were 1.95 *P* 0.0057, 1.77 *P* 0.0014, 1.98 *P* < 0.0001, 2.41 *P* <0.0001, respectively (for all CMT1A to all controls FC 2.07 *P* < 0.0001). For TMPRSS5, none of the individual bins were significantly different from each other. For NfL, FC 1.11 *P* 0.9973, 1.38 *P* 0.2048, 1.57 *P* 0.008, 1.68 *P* 0.0001, 2.19 *P* < 0.0001, respectively (all CMT1A to all controls FC1.58 *P* < 0.0001). For Nfl only, the CMT1A > 20 bin was significantly elevated compared to the <5 bin FC 1.98 *P* 0.03, out of all the comparisons of the bins to each other. Statistically significant elevation in comparisons of CMT1A cohorts to control cohorts is summarized as indicated ****P < 0.0001, ***P 0.0001–0.003, **P 0.0014–0.008, *0.0194, not significant (ns) >0.20.

**Figure 4:**
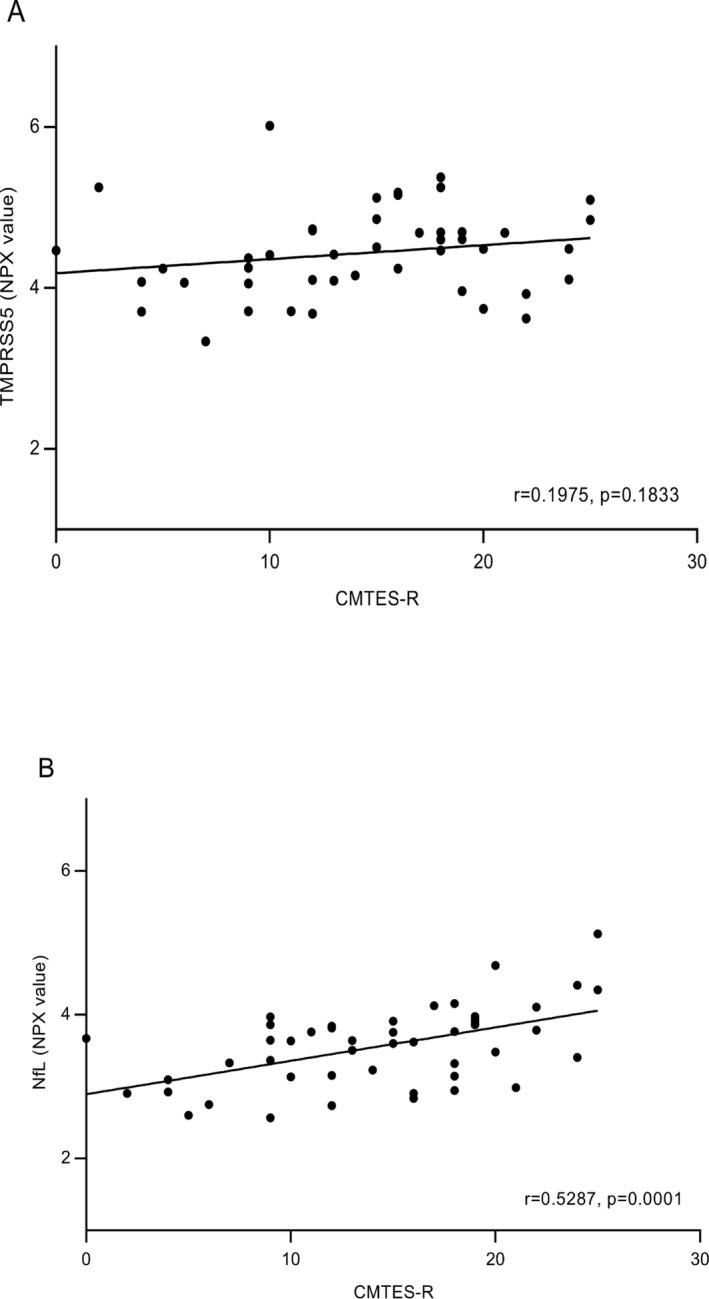
Pearson Correlation of CMT1A TMPRSS5 and NfL levels with Neuropathy Scores Unbinned. Correlation of TMPRSS5 (A) and NfL (B) levels with CMTES‐R neuropathy scores are shown, with *r* and *P* values. Following age correction by partial correlation using the data from Figure 2, NfL correlation with CMTES‐R *r* = 0.38, *P* = 0.081 (NfL with CMTNS‐R *r* = 0.3695 *P* = 0.0174).

The correlation of severity of neuropathy as assessed by the Rasch‐modified CMTES score with TMPRSS5 and NfL CMT1A patient data from Figure 1 was determined. First, we used a binned analysis in which mild (CMTES‐R < 5), moderate (CMTES‐R 6–10), and more severe cases (11–15 and 16–20) were grouped (Fig. 3). For TMPRSS5 although all bins showed significant upregulation of comparable magnitude fold change (1.95‐ to 2.41‐fold) compared to all controls, no significant correlation with CMTES‐R was observed as none of the bins were statistically different *P* < 0.05 from each other (Fig. 3A). Second, the correlation between CMTES‐R and TMPRSS5 for all samples was determined (Fig. 4A) and was not significant (*r* = 0.1975 *P* = 0.1833). The same pattern of upregulation of TMPRSS5 was seen in all the equivalent CMTNS‐R score bins, and no significant correlation with CMTNS‐R (all samples) was observed (*r* = 0.22 *P* = 0.15 data not shown). This suggests TMPRSS5 is chronically elevated in the progression of CMT1A. No significant correlation was observed between TMPRSS5 and the electrophysiological parameters CMAP and MNCV (all CMT1A samples *r* = −0.12 *P* = 0.45 and *r* = −0.22 *P* = 0.158, respectively – data not shown). As expected, following age correction, no correlation was observed between TMPRSS5 and CMTES‐R, CMTNS‐R, Ulnar CMAP, or Ulnar MNCV (data not shown).

In contrast, NfL, as expected,^19^ increased across the CMTES‐R score bins (Fig. 3B). The two lowest score bins CMTES‐R < 5 and 5 < 10 were not significantly different from the controls (1.11‐fold, *P* = 0.9973; 1.38‐fold *P* = 0.2048, respectively), whereas the top three score bins were significantly higher (1.57‐fold *P* = 0.008, 1.68‐fold 0.0001, 2.19‐fold <0.0001, respectively). Also, the top score bin CMTES > 20 was significantly upregulated compared to the lowest score bin CMTES < 5 (1.98‐fold, *P* = 0.03). There was a significant correlation between CMTES‐R and NfL when plotted for all samples (*r* = 0.53 *P* = 0.0001) (Fig. 4B). The same pattern of increase in NfL was seen across CMTNS‐R score bins, and a significant correlation was observed with all samples (*r* 0.54, *P* = 0.003 data not shown). Following age correction, the correlation of NfL with CMTES‐R and CMTNS‐R remained significant but with reduced *r* values (*r* = 0.38 *P* = 0.0081 and *r* = 0.3695 *P* = 0.0174, respectively). No significant correlation was observed between NfL and the electrophysiological parameter CMAP (all CMT1A samples *r* = 0.19 *P* = 0.24, after age correction *r* = 0.22 *P* = 0.168). An apparent positive correlation with NCV (all CMT1A samples *r* = 0.32 *P* = 0.0418) was not significant following age correction (*r* = 0.29 *P* = 0.070).

### Specificity for CMT1A

**Figure 5:**
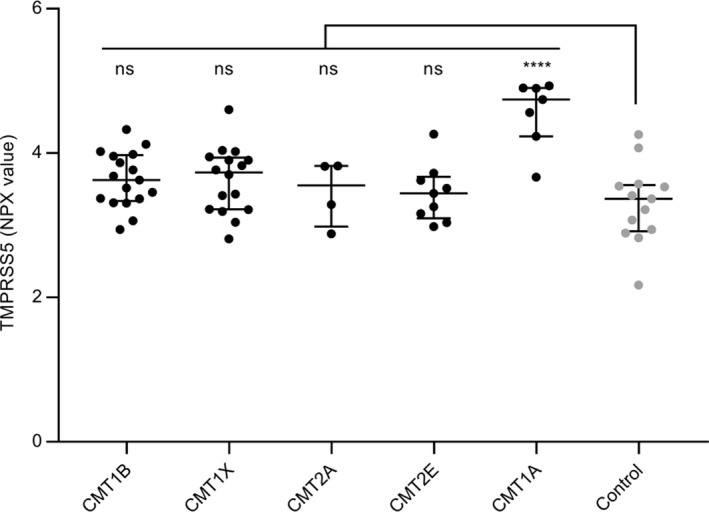
Evaluation of TMPRSS5 in plasma samples from different CMT subtypes. TMPRSS5 levels in plasma samples from CMT1B (*n* = 17), CMT1X (*n* = 16), CMT2A (*n* = 4), CMT2E (*n* = 9), CMT1A (*n* = 7) compared to control (*n* = 13) shown as NPX with median and IQR. FC and *P* (Abnova comparison of groups followed by Dunnett’s multiple comparison test) for comparison with controls were CMT1B 1.26 *P* 0.1876, CMT1X 1.26 *P* 0.2003, CMT2A 1.11 *P* 0.9647, CMT2E 1.11 *P* 0.9156, CMT1A 2.40 *P* 0.0001. Statistically significant elevation to the control cohort is indicated ****P 0.0001, not significant (ns) >0.20.

CMT1A is the prototype of the demyelinating form of CMT, so we performed a pilot Olink Neurology panel analysis of samples obtained from two other types of demyelinating CMT: CMT1B caused by mutations in the *Myelin Protein Zero* gene,^30^ and CMT1X, caused by mutations in the *Gap Junction beta1* gene.^31^ For comparison, we also included samples from two other axonal forms of CMT: CMT2A, caused by mutations in the *Mitofusin 2* gene^32^ and CMT2E caused by mutations in the *NEFL* gene.^33^ The level of TMPRSS5 was determined for a subset of CMT1A and samples of the other CMT subtypes and controls (Fig. 5). The expected increase in TMPRSS5 in CMT1A was observed (average FC 2.4 *P* = 0.0001), but only a modest increase was seen for CMT1B and CMT1X samples (demyelinating forms) which was not statistically significant (FC 1.26 *P* = 0.1876 and FC 1.26 *P* = 0.2003, respectively). No significant increase was seen for CMT2A and CMT2E (axonal forms) (FC 1.11 *P* = 0.9647 and FC 1.11 *P* = 0.9156, respectively). Therefore, the elevation of TMPRSS5 may be specific to CMT1A and not elevated in all forms of CMT. As there is a wide range of different specific mutations with a range of disease severity in CMT1B and CMT1X, it is possible that patients with specific mutations within these subtypes may prove to have elevated TMPRSS5 levels. Further testing of additional patient sample with known specific mutations would be needed to investigate this.

### Schwann cell‐specific expression of TMPRSS

**Figure 6:**
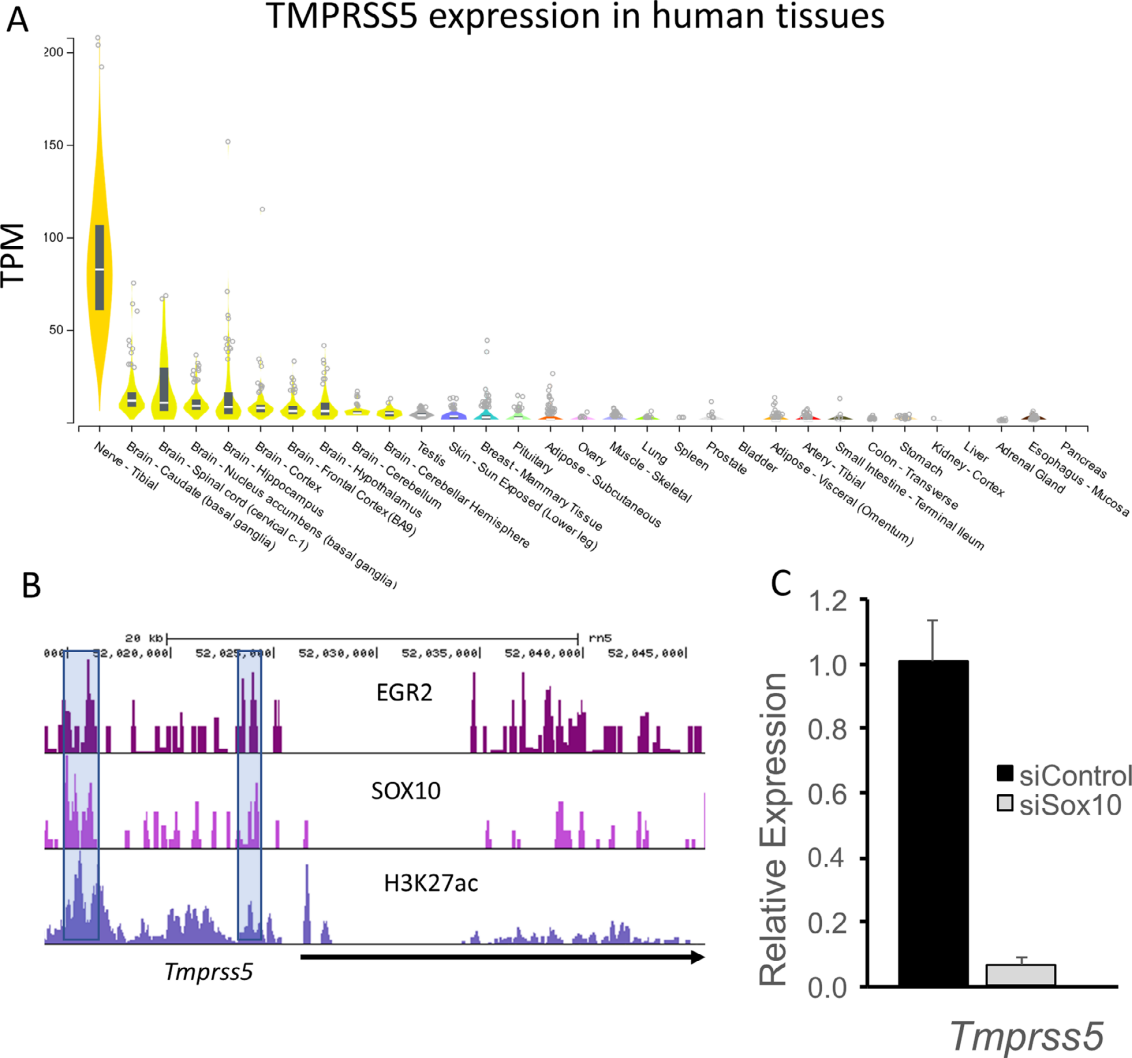
Specificity and Regulation of TMPRSS5 expression. (A) Human tissue profiling data (gtexportal.org) show highest expression of TMPRSS5 in peripheral nerve (tibial nerve), which is predominantly composed of Schwann cells. Lower expression levels are found in different brain regions. (B) The panel shows ChIP‐seq analysis around the Tmprss5 gene in rat peripheral nerve, and the shaded bars indicate upstream binding sites for two Schwann cell transcription factors (EGR2 and SOX10), along with colocalized histone H3K27 acetylation, which is a marker of engaged enhancers. (C) To determine regulation of TMPRSS5, siRNA directed against Sox10 was transfected into the S16 rat Schwann cell line. The results show dramatic downregulation of Tmprss5 transcript with Sox10 depletion, as Sox10 is required for expression of many myelin genes in Schwann cells.

The tissue‐specific expression of *TMPRSS5* in Schwann cells was evaluated in several ways. First, profiling of human tissues performed by the Broad Institute (gtexportal.org) identified the highest levels in human peripheral nerve (i.e., tibial nerve) (Fig. 6A). Lower amounts are observed in salivary gland and some CNS tissues, but expression of *TMPRSS5* was almost negligible in other tissues. Consistent with these data, cell sorting studies in mouse skin showed that expression of *Tmprss5* was confined to Schwann cells.^34^ Many myelin‐associated genes are regulated by Sox10 expression, which is an important transcription factor at all stages of Schwann cell development. We had previously reported on Sox10‐dependent genes in the S16 Schwann cell line,^35^ and found that Tmprss5 is regulated by Sox10, which was confirmed by independent quantitative RT‐PCR analysis. Sox10 binding by ChIP‐seq was analyzed in both peripheral nerve and spinal cord,^36^ and shows binding of Sox10 to enhancers that are marked by the presence of histone H3K27 acetylation (Fig. 6B). Notably, there is strong Sox10 binding in both oligodendrocytes and Schwann cells to an enhancer located ~11 kb upstream of the gene in the rat genome. Next, we performed a Sox10 RNAi knockdown experiment in cultured S16 rat Schwann cells and demonstrated a substantial, over 90% reduction, in TMPRSS5 mRNA levels (Fig. 6C). Finally, after nerve injury, many SC‐specific myelin genes like PMP22 decline dramatically as Schwann cells lose contact with degenerating axons, and then they are reactivated if nerve regeneration occurs. The Tmprss5 gene, similarly declines after nerve injury and then increases again as regeneration/remyelination occurs, indicating that TMPRSS5 is regulated by the Schwann cell differentiation program.^37^, ^38^, ^39^

## Discussion

The analysis of independent cohorts of CMT1A plasma samples and controls by Olink assays (total 398 proteins) identified two proteins, TMPRSS5 and NfL, that were consistently increased >average FC 2.07 and 1.58, respectively, in subjects with CMT1A. The elevation of TMPRSS5 in CMT1A was also confirmed with an orthogonal Imperacer Immuno‐PCR assay.

TMPRSS5 was consistently elevated about twofold across all CMT1A patients but was not correlated to severity of neuropathy by CMTES‐R or CMTNS‐R, or to electrophysiological parameters CMAP and MNCV, or to age. It was elevated very early in disease, even at the lowest CMTES‐R or CMTNS‐R scores, and was consistently elevated throughout the progression of the disease as measured across the higher disease scores. Hence, TMPRSS5 is indicated to be chronically and consistently elevated in CMT1A with an AUC of 0.9131. It was not significantly elevated in CMT1B, CMT1X (demyelinating CMTA forms) or CMT2A, CMT2E (axonal CMT forms), indicating it may be a biomarker specific to CMT1A, and not involved in the pathogenesis of other forms of CMT. It was not correlated to NfL or to age. From our analysis of multiple CMT1A and normal cohorts, TMPRSS5 and NfL are indicated to have long‐term multi‐year stability in samples stored at −80°C. Further studies are required to determine the stability of TMPRSS5 within CMT1A patients with longitudinal samples. A steady‐state elevation of a biomarker of a disease process can be quite useful if it proves responsive to treatment. This has been noted in previous reviews.^40^

In contrast, for NfL, we found that it correlated with age (*r* = 0.47 controls), and with CMTES‐R (*r* = 0.37) and CMTNS (*r* = 0.37) after age correction. It did not correlate with electrophysiological parameters CMAP and MNCV after age correction. In the previously reported study of CMT subtype samples, including CMT1A and 11 other subtypes (31 out of the total 75 patient samples were CMT1A),^19^ the combined data for all subtypes also showed NfL increased with age (*r* 0.7 controls) and with CMTES (*r* 0.41) after age correction and so significant correlation was found with CMAP. NfL is indicated to be a good biomarker of axonal degeneration in CMT1A and other CMT subtypes, as well as a range of neurological diseases including ALS,^41^ frontal temporal dementia,^42^ and MS.^43^ NfL may prove a good efficacy biomarker in CMT; interestingly, NfL plasma levels were elevated in a mouse model of CMT4C and were returned to normal with successful gene therapy treatment.^44^ Further studies are required to determine its stability in CMT1A longitudinal samples. Its correlation with age must be considered in clinical trials and correction for age effect may be necessary.

TMPRSS5 (also known as spinesin) contains a transmembrane domain, a stem region containing a scavenger receptor‐like domain, and a serine protease domain that is able to cleave trypsin substrates.^28^ However, no knockout mouse has been characterized, and its specific role in CMT1A or regulation of myelination remains unknown. Mutations in *TMPRSS5* have been associated with deafness,^41^ and other family members may play similar roles. Interestingly, several types of CMT including CMT1A are associated with hearing loss.^42^, ^43^, ^44^

TMPRSS5 is highly expressed in human peripheral nerve with some lower level expression in salivary gland and the CNS (gtexportal.org). In a mouse database of cell type‐specific expression in skin,^34^ TMPRSS5 showed preferential expression in Schwann cells, presumably due to its regulation by the Sox10 transcription factor.^35^. Furthermore, in the S16 rat Schwann cell line, depletion of Sox10 reduced TMPRSS5 level, indicating that Tmprss5, like many other myelin genes, is regulated by this critical transcription factor required for Schwann cell development.^35^ While it is not yet clear that increased TMPRSS5 levels in plasma are due to enhanced release from Schwann cells and/or increased synthesis, the Tmprss5 transcript is elevated in a recently published profile of the rat CMT1A model,^45^ and analysis of the C3 model of CMT1A showed a similar elevation of the Tmprss5 transcript.

The levels of TMPRSS5 are consistently elevated in CMT1A samples with a good discrimination of cases versus controls, but there is no statistically significant correlation with either neuropathy score or age. This would be expected in a protein whose expression is mechanistically correlated with impaired Schwann cell myelination. The decreased nerve conduction velocities associated with impaired myelination are evident early in the course of CMT1A, and do not further decrease with the progression of the disease, indicating that the level of demyelination is relatively constant. Studies of patients with CMT1A^46^ and rodent models^47^, ^48^ demonstrate that impairment correlates more with axonal degeneration than demyelination in CMT1A.

Taken together, we believe our results for TMPRSS5 and NfL support a complementary biomarker strategy in CMT1A in which TMPRSS5 can serve as a novel potential biomarker for myelinating Schwann cells, along with assessments of NfL as a marker for axonal degeneration. Key additional studies are required to look at the longitudinal stability of these biomarkers in patients, and to determine if they decrease in appropriate animal efficacy studies and hence could be used as efficacy biomarkers.

## Authors’ Contributions

KW, HW, MD, MS, and JS were responsible for study concept and design. HW, MD, JS, and MS drafted the manuscript and figures. KW, HW, MD, TX, MK, KC, JL, XW, RZ, CB, YB, JM, LG, DA, AR, MR, JS, and MS performed data acquisition and analysis.

## Conflict of Interest

There are no conflicts of interest.

## Supporting information


**Figure S1**. Elevation of TMPRSS5 and 3 other proteins in initial pilot experiment with CMT1A and controls.

**Figure S2**. Increase in TMPRSS5 and NfL confirmed by orthogonal assays.
