# Supplementary figures and images for: Transmembrane protease serine 5: a novel Schwann cell plasma marker for CMT1A
Source: Ann Clin Transl Neurol. 2019 Dec 12;7(1):69–82. doi: 10.1002/acn3.50965 (PMC6952315; doi:10.1002/acn3.50965)

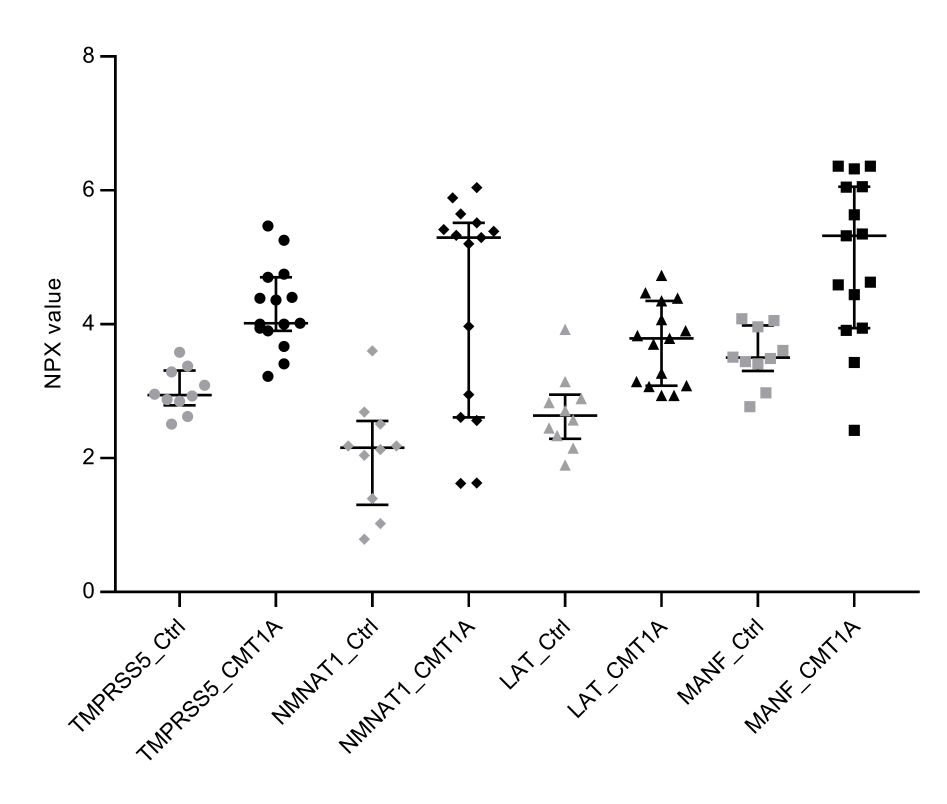

Supplement: Supplementary file 1 — Figure S1. Elevation of TMPRSS5 and 3 other proteins in initial pilot experiment with CMT1A and controls. [file ACN3-7-69-s001.tiff]

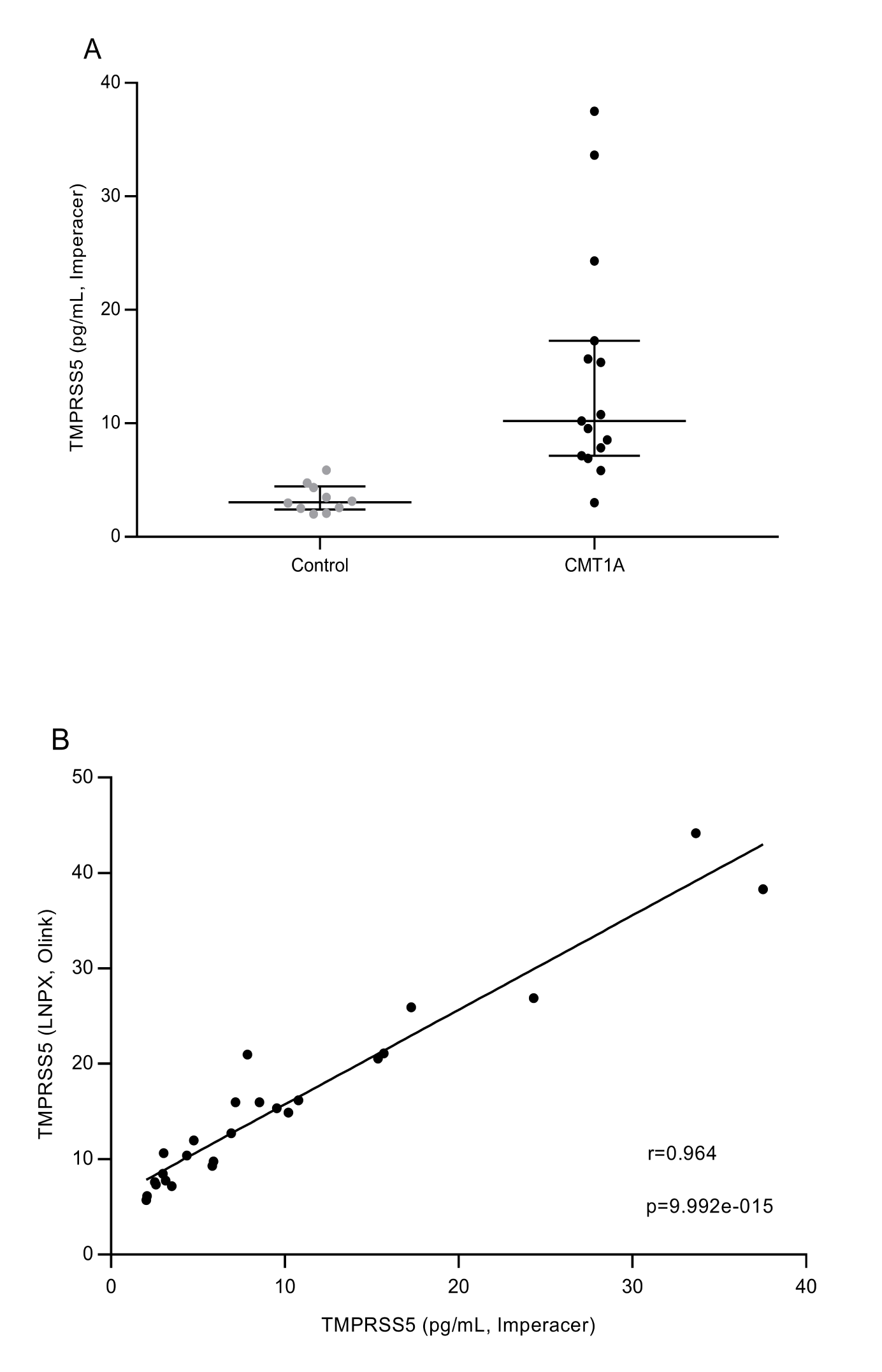

Supplement: Supplementary file 2 — Figure S2. Increase in TMPRSS5 and NfL confirmed by orthogonal assays. [file ACN3-7-69-s002.tiff]
